# Hot Deformation Behavior and a Two-Stage Constitutive Model of 20Mn5 Solid Steel Ingot during Hot Compression

**DOI:** 10.3390/ma11030434

**Published:** 2018-03-16

**Authors:** Min Liu, Qing-Xian Ma, Jian-Bin Luo

**Affiliations:** Department of Mechanical Engineering, Tsinghua University, Beijing 100084, China; liumin881120@126.com (M.L.); luojblqw@mail.tsinghua.edu.cn (J.-B.L.)

**Keywords:** 20Mn5 steel, hot deformation behavior, constitutive model, heavy forging

## Abstract

20Mn5 steel is widely used in the manufacture of heavy hydro-generator shaft forging due to its strength, toughness, and wear resistance. However, the hot deformation and recrystallization behaviors of 20Mn5 steel compressed under a high temperature were not studied. For this article, hot compression experiments under temperatures of 850–1200 °C and strain rates of 0.01 s^−1^–1 s^−1^ were conducted using a Gleeble-1500D thermo-mechanical simulator. Flow stress-strain curves and microstructure after hot compression were obtained. Effects of temperature and strain rate on microstructure are analyzed. Based on the classical stress-dislocation relationship and the kinetics of dynamic recrystallization, a two-stage constitutive model is developed to predict the flow stress of 20Mn5 steel. Comparisons between experimental flow stress and predicted flow stress show that the predicted flow stress values are in good agreement with the experimental flow stress values, which indicates that the proposed constitutive model is reliable and can be used for numerical simulation of hot forging of 20Mn5 solid steel ingot.

## 1. Introduction

Heavy cylinder forgings are widely used in key equipment such as a nuclear pressure vessel, hydro-generator shaft, and hydrogenation reactor [1,2].

20Mn5 steel has been widely used in the manufacture of hydro-generator shaft forging due to its good balance of strength, toughness, wear resistance, and weldability. However, for heavy 20Mn5 solid steel ingot, defects including coarse grains and shrinkage cavities exist. To lower the cost of production and shorten the production cycle of the heavy hydro-generator shaft forging, it is necessary to carry out numerical simulations to obtain reasonable forging process parameters to make sure that the coarse grains can be refined and shrinkage cavities can be eliminated. In order to carry out a successful simulation of the hot forging process, a precise constitutive model that describes the effect of temperature, strain rate, and strain on stress is essential. At present, there are no reports on the constitutive model of heavy 20Mn5 solid steel ingot.

In this article, hot compression experiments of 20Mn5 steel under temperatures of 850–1200 °C and strain rates of 1/s, 0.1/s, and 0.01/s are carried out using a Gleeble-1500D thermo-mechanical simulator. Flow stress-strain curves and microstructure after hot compression on different temperatures and strain rates are obtained. Based on the experimental flow stress curves, a two-stage constitutive model is established by introducing the classical stress–dislocation relationship and the kinetic equation of dynamic recrystallization (DRX) [3,4,5,6,7,8,9,10,11,12,13,14,15,16,17,18,19,20,21,22]. By comparisons of the predicted flow stress values and experimental flow stress values, the proposed physically-based constitutive model shows high accuracy. Therefore, the newly developed constitutive model of 20Mn5 steel can be used for numerical simulations of the hot forging process. Moreover, the flow stress curves and microstructure after hot compression can provide an important reference for the establishment of forging process specifications for a 20Mn5 heavy hydro-generator shaft.

## 2. Materials and Methods

### 2.1. Experimental Material

**Figure 1:**
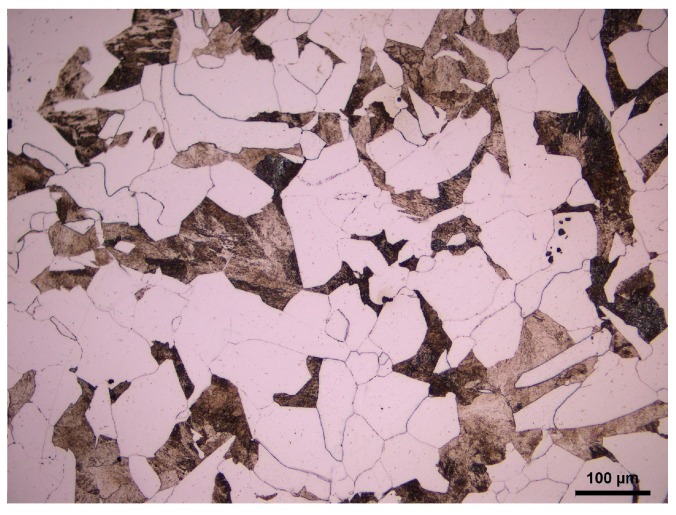
The initial as-cast microstructure of 20Mn5 steel.

Cylindrical samples with a diameter of 8 mm and a height of 12 mm were taken from a 20Mn5 solid steel ingot used for a heavy hydro-generator shaft. The chemical composition of 20Mn5 steel is given in Table 1. Figure 1 shows the initial as-cast microstructure of 20Mn5 steel, which contains ferrite and pearlite. The white part represents ferrite, and the black part represents pearlite.

### 2.2. Experimental Procedure

The hot compression experiments were carried out on a Gleeble-1500D (DSI, New York, NY, USA) thermal and mechanical simulation machine. First, the specimens were heated to 1000 °C at a heating rate of 5 °C/s and held there for 3 min. Then, the temperature was adjusted to deformation temperature (850–1200 °C, with a 50 °C interval) at 10 °C/s and held for 60 s to get a uniform temperature distribution. Then, the specimens were compressed to a true strain of 0.7 under deformation temperatures of 850–1200 °C with a 50 °C interval and strain rates of 1/s, 0.1/s, and 0.01/s. Finally, the specimens were quenched with water in order to retain the morphologies of austenite grains. After the hot compression experiments, the specimens were cut, ground, polished, and etched in the mixture of 5 g picric acid, 4 g sodium dodecyl benzene sulfonate, and 100 mL water at 60–70 °C. Then, the morphologies of emerged austenite grains were observed by OLYMPUS BX51 microscope (Olympus Corporation, Tokyo, Japan).

## 3. Results and Discussion

### 3.1. Flow Stress Curves

**Figure 2:**
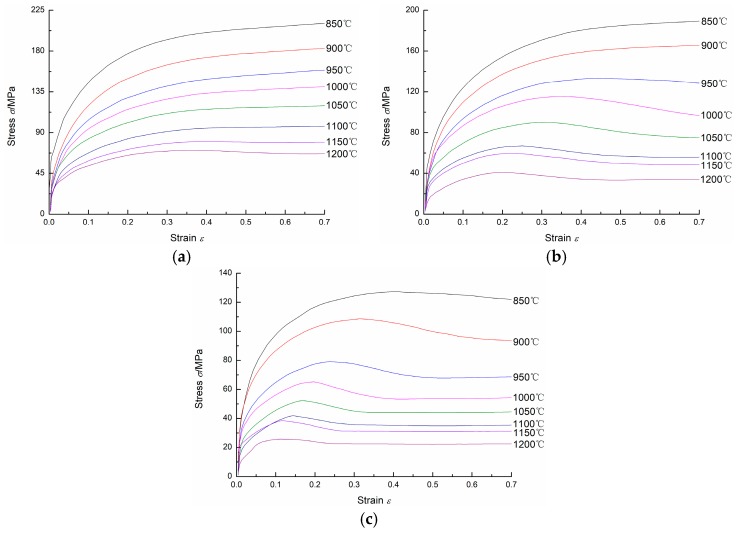
Flow stress curves of the 20Mn5 steel under strain rates of: (**a**) 1/s; (**b**) 0.1/s; (**c**) 0.01/s.

Figure 2 shows the flow stress curves of 20Mn5 steel compressed at different temperatures and different strain rates. As shown in Figure 2, for the same strain rate and strain, the flow stress is larger when the specimen is compressed at a lower temperature; for the same temperature and strain, the flow stress is larger when the specimen is compressed at higher strain rate. It can easily be seen that at high temperatures and low strain rates, the flow stress increases to a peak stress at first, and then reduces to a steady-state stress and remains as a constant as the strain increases. For low temperatures and high strain rates, the steady-state stress does not occur. Moreover, for the same strain rate, the peak strain and steady-state strain increase when the compression temperature decreases; for the same temperature, the peak strain and steady-state strain increase when the strain rate increases.

For 20Mn5 steel, the flow stress is influenced by work hardening (WH), dynamic recovery (DRV), and dynamic recrystallization (DRX), which compete during hot compression. In the initial deformation stage, working hardening and dynamic recovery occur. Dislocation movement and rearrangement can reduce the dislocation density, but working hardening comes into prominence. As a result, the flow stress increases as the strain increases. When strain reaches a critical value, dynamic recrystallization occurs and the softening effect caused by dynamic recrystallization dominates. Correspondingly, the flow stress increases gradually until peak stress at first, and then decreases as the strain increases. Finally, a dynamic balance between working hardening, dynamic recovery, and dynamic recrystallization is reached. The flow stress remains constant as the strain increases [23,24].

### 3.2. Effect of Temperature and Strain Rate on Microstructure after Hot Compression

**Figure 3:**
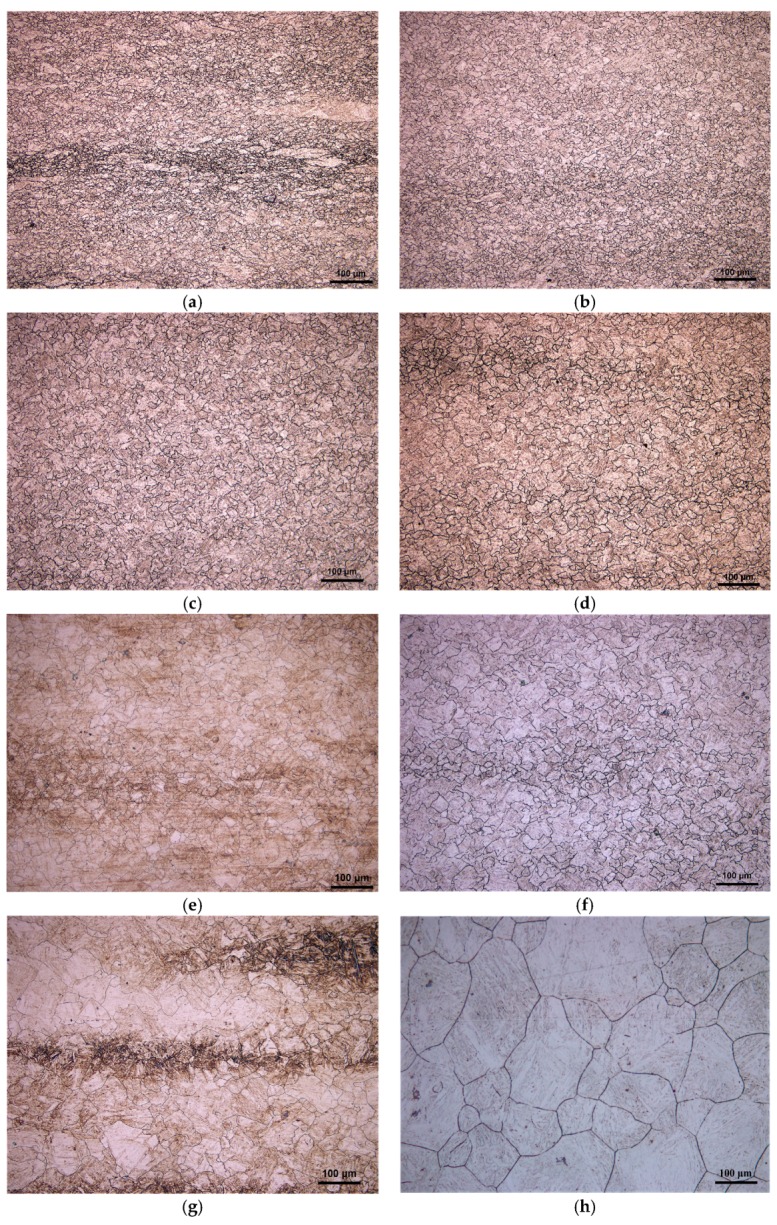
Microstructure of 20Mn5 steel compressed to a true strain of 0.7 under strain rate of 0.01/s and temperatures of: (**a**) 850 °C; (**b**) 900 °C; (**c**) 950 °C; (**d**) 1000 °C; (**e**) 1050 °C; (**f**) 1100 °C; (**g**) 1150 °C; (**h**) 1200 °C.

Figure 3 shows the microstructure of 20Mn5 steel compressed to a true strain of 0.7 under a strain rate of 0.01/s and different temperatures. It is obvious that the austenite grain size increases with the increase of the compression temperature. Under eight temperatures and a strain rate of 0.01/s, dynamic recrystallization occurs. The occurrence of dynamic recrystallization shown in Figure 3 can also be seen from the measured flow stress curves. In the above flow stress curves corresponding to Figure 3, the flow stress curves all reach a steady state when the strain equals a certain value, which indicates that dynamic recrystallization is complete.

**Figure 4:**
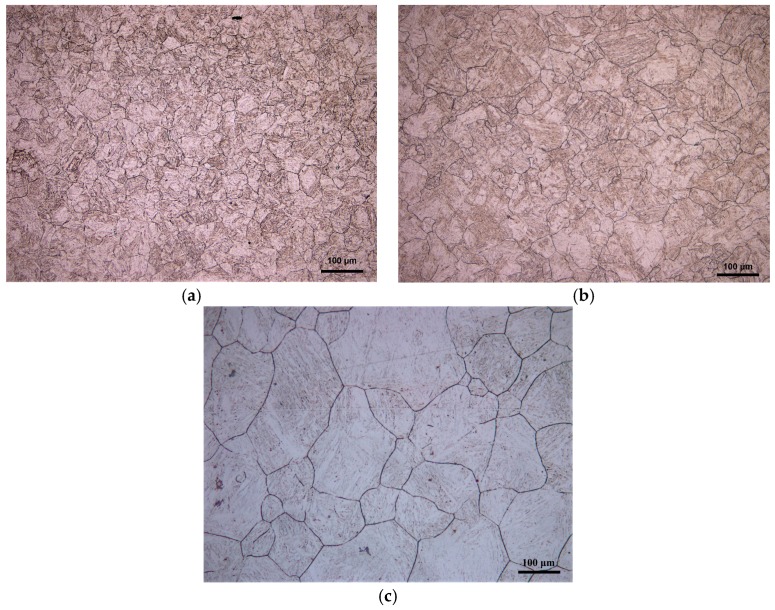
Microstructure of 20Mn5 steel compressed to a true strain of 0.7 under temperature of 1200 °C and strain rates of: (**a**) 1/s; (**b**) 0.1/s; (**c**) 0.01/s.

Figure 4 shows the microstructure of 20Mn5 steel compressed to a true strain of 0.7 at 1200 °C and different strain rates. The higher the strain rate, the smaller the grain size. The reason for this phenomenon is that the high strain rate reduces the deformation time at a certain strain and the recrystallized nucleus has insufficient time to fully grow [23,24].

## 4. Establishment of a Two-Stage Constitutive Model and Its Verification

### 4.1. The Derivation of a Two-Stage Constitutive Model

**Figure 5:**
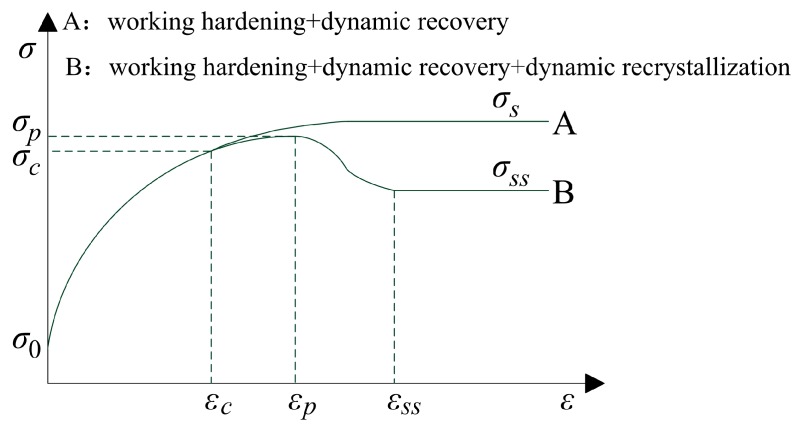
Typical flow stress curves for hot working.

Figure 5 shows two typical flow stress curves for hot working. In curve B, in the initial deformation stage (0 ≤ *ε* < *ε_c_*), stress increases from *σ*_0_ to *σ_c_* as a result of working hardening and dynamic recovery. When strain equals a critical value *ε_c_*, dynamic recrystallization occurs. As strain increases, first, the stress increases from *σ_c_* to *σ_p_*, and then stress decreases from *σ_p_* to a steady-state stress *σ_ss_*. If dynamic recrystallization never occurs in some materials, the change of stress with strain will be similar to curve A.

The flow stress curve of 20Mn5 steel in this study is similar to curve B, and a two-stage method is used to establish the flow stress model of 20Mn5 steel.

(1) Stage of working hardening and dynamic recovery (0 ≤ *ε* < *ε_c_*)

The evolution of the dislocation density with strain is generally considered to be as follows:

(1) $$\frac{d\rho }{d\varepsilon }=k_{1}\sqrt{\rho }-k_{2}\rho ,$$

where *k*_1_ represents the coefficient of working hardening and *k*_2_ is the coefficient of dynamic recovery.

When ε=0, $\rho =\rho_{0}$, where $\rho_{0}$ is the initial dislocation density.

By integration of Equation (1), dislocation density ρ can be expressed as follows:

(2) $$\rho ={\left(\frac{k_{1}}{k_{2}}-\frac{k_{1}}{k_{2}}e^{-\frac{k_{2}}{2}\varepsilon }+\sqrt{\rho_{0}}e^{-\frac{k_{2}}{2}\varepsilon }\right)}^{2},$$

when $\frac{d\rho }{d\varepsilon }=0$, $\rho_{s}={\left(\frac{k_{1}}{k_{2}}\right)}^{2}$, where $\rho_{s}$ is the saturation dislocation density corresponding to the saturation stress $\sigma_{s}$.

Based on the above equations and the classical Taylor relation $\sigma =\alpha \mu b\sqrt{\rho }$, where α is the material constant, μ is the shear modulus, and b is the distance between atoms in the slip direction, the stress $\sigma_{WH}$ at strain ε can be expressed as follows:

(3) $$\sigma_{WH}=\sigma_{s}+(\sigma_{0}-\sigma_{s})e^{-\frac{k_{2}}{2}\varepsilon },\left(\varepsilon ＜\varepsilon_{c}\right).$$

(2) Stage of dynamic recrystallization (*ε* ≥ *ε_c_*)

The volume fraction of DRX, $X_{drx}$, can be determined by the following equation:

(4) $$X_{drx}=1-\exp(-k_{d}{\left(\frac{\varepsilon -\varepsilon_{c}}{\varepsilon_{p}}\right)}^{n_{d}}),\;\left(\varepsilon \geq \varepsilon_{c}\right),$$

where $X_{drx}$ is the volume fraction of DRX, $\varepsilon_{c}$ is the critical strain, $\varepsilon_{p}$ is the peak strain, and $k_{d}$ and $n_{d}$ are constants related to material.

The relationship between $X_{drx}$ and stress parameters can be given as follows:

(5) $$X_{drx}=\frac{\sigma_{WH}-\sigma }{\sigma_{s}-\sigma_{ss}},\;\left(\varepsilon \geq \varepsilon_{c}\right),$$

where $\sigma_{s}$ is the saturation stress, $\sigma_{ss}$ is the steady-state stress, $\sigma_{WH}$ is the stress at strain ε calculated by Equation (3), and σ is flow stress at strain ε.

By combining Equations (4) and (5), the flow stress during DRX period can be given by the following expression:

(6) $$\sigma =\sigma_{WH}-(\sigma_{s}-\sigma_{ss})(1-\exp(-k_{d}{\left(\frac{\varepsilon -\varepsilon_{c}}{\varepsilon_{p}}\right)}^{n_{d}})),\;\left(\varepsilon \geq \varepsilon_{c}\right).$$

### 4.2. Determination of Material Constants (α, n, Q, and A) Based on the Peak Stress

The Arrhenius equation proposed by Sellars and Tegart is widely used to describe the relationship between flow stress, strain rate and temperature:

(7) $$\overset{•}{\varepsilon }=A{\left[\sinh(\alpha \sigma )\right]}^{n}\exp(-\frac{Q}{RT}),$$

where A, α, and n are material constants; Q is the activation energy of deformation (J/mol); $\overset{•}{\varepsilon }$ is the strain rate (s^−1^); σ is the flow stress (MPa); T is the absolute temperature (K); and R is the gas constant (8.314 J/(mol·K)).

Based on a Taylor expansion, the following equations can be obtained:

(8) $$e^{\alpha \sigma }=1+\alpha \sigma +\frac{{\left(\alpha \sigma \right)}^{2}}{2!}+\frac{{\left(\alpha \sigma \right)}^{3}}{3!}+\ldots ,$$

(9) $$e^{-\alpha \sigma }=1+(-\alpha \sigma )+\frac{{\left(-\alpha \sigma \right)}^{2}}{2!}+\frac{{\left(-\alpha \sigma \right)}^{3}}{3!}+\ldots ,$$

(10) $$\sinh(\alpha \sigma )=\frac{e^{\alpha \sigma }-e^{-\alpha \sigma }}{2}=\alpha \sigma +\frac{{\left(\alpha \sigma \right)}^{3}}{3!}+\ldots ,$$

According to Equations (7) and (10), the following equations can be easily obtained:

(11) $$\overset{•}{\varepsilon }=A_{1}\sigma^{n_{1}}\exp(-\frac{Q}{RT}),\;\mathrm{for}\;\mathrm{low}\;\mathrm{stress}\;\mathrm{level}$$

(12) $$\overset{•}{\varepsilon }=A_{2}\exp(\beta \sigma )\exp(-\frac{Q}{RT}),\;\mathrm{for}\;\mathrm{high}\;\mathrm{stress}\;\mathrm{level}.$$

According to Equations (7), (11) and (12), the following equation is obvious:

(13) $$\alpha =\frac{\beta }{n_{1}}.$$

Taking the natural logarithms on both sides of Equations (7), (11), and (12), the following expressions can be obtained:

(14) $$\ln\overset{•}{\varepsilon }=\ln A+n\ln[\sinh(\alpha \sigma )]+(-\frac{Q}{RT}),$$

(15) $$\ln\overset{•}{\varepsilon }=\ln A_{1}+(-\frac{Q}{RT})+n_{1}\ln\sigma ,$$

(16) $$\ln\overset{•}{\varepsilon }=\ln A_{2}+(-\frac{Q}{RT})+\beta \sigma .$$

**Figure 6:**
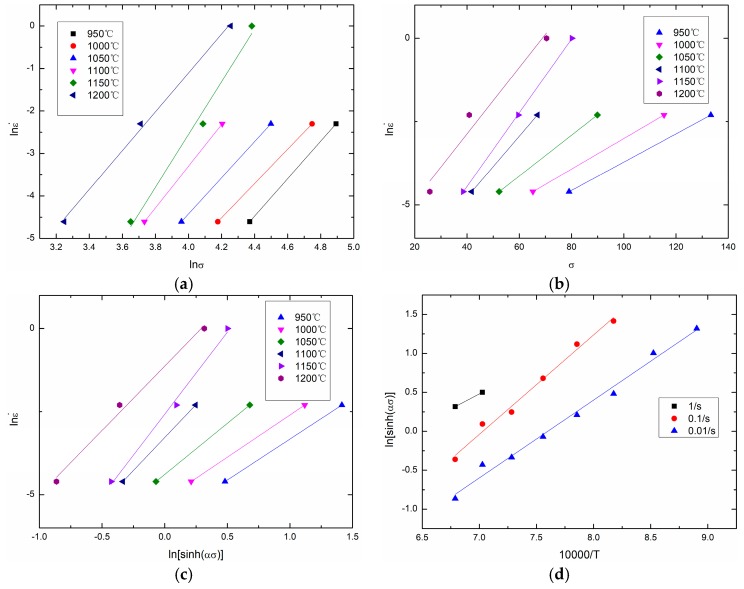
Calculation of material constants: (**a**) *n*_1_; (**b**) *β*; (**c**) *n*; (**d**) *Q* and *A*.

In this study, σ in the above equations is taken as the peak stress $\sigma_{p}$. Based on experimental data and Equations (14)–(16), Figure 6 is drawn as follows. In Figure 6a, the slopes of the lines equal *n*_1_, and *n*_1_ = 4.724533 is obtained. In Figure 6b, the slopes of the lines equal *β*, and *β* = 0.075217 is obtained. Thus, *α* = *β*/*n*_1_ = 0.01592. In Figure 6c, the slopes of the lines equal *n*, and *n* = 3.474378 is obtained. In Figure 6d, the slopes of the lines equal *Q*/(10,000 nR), and the intercepts of the lines equal −(1/*n*)lnA + (1/*n*)ln$\overset{•}{\varepsilon }$. Thus, *Q* = 293561.4895 J/mol and *A* = 1.13 × 10^12^ can be obtained.

From Equation (7), the following equation can be obtained:

(17) $$\sigma_{p}=\frac{\ln({\left(\frac{Z}{A}\right)}^{\frac{1}{n}}+\sqrt{{\left(\frac{Z}{A}\right)}^{\frac{2}{n}}+1})}{\alpha }=\frac{\ln({\left(\frac{\overset{\cdot }{\varepsilon }\exp(\frac{Q}{RT})}{A}\right)}^{\frac{1}{n}}+\sqrt{{\left(\frac{\overset{\cdot }{\varepsilon }\exp(\frac{Q}{RT})}{A}\right)}^{\frac{2}{n}}+1})}{\alpha }.$$

Based on the calculated material constants (α, n, Q, and A), the peak stress $\sigma_{p}$ at any temperature T and any strain rate $\overset{•}{\varepsilon }$ can be obtained.

### 4.3. Determination of Materials Parameters (ε_p_, ε_c_, σ_s_, σ_ss_, σ_0_, k_2_, k_d_, n_d_) in the Two-Stage Constitutive Model

The peak strain $\varepsilon_{p}$ can be obtained directly from the experimental flow stress curves. The mathematical model of $\varepsilon_{p}$ can be expressed as follows:

(18) $$\varepsilon_{p}=kZ^{m}.$$

**Figure 7:**
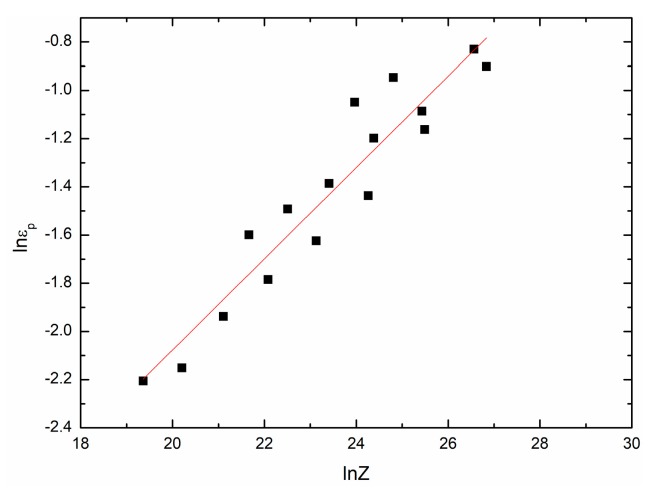
The relationship between $\ln\varepsilon_{p}$ and lnZ.

By taking natural logarithm on both sides of Equation (18) and using different groups of $\varepsilon_{p}$, $\overset{•}{\varepsilon }$, T, and Q, Figure 7 can be drawn as follows. According to the fit result in Figure 7, $\varepsilon_{p}$ can be expressed as a function of *Z*:

(19) $$\varepsilon_{p}=0.0028429Z^{0.18926}.$$

The critical strain and critical stress can be obtained from the θ–σ (working hardening rateθ=dσ/dε) curve. Kim et al. propose that the θ–σ curve can be divided into three segments. The first segment starts from $\sigma_{0}$ and ends with $\sigma_{c}$, during which working hardening and dynamic recovery exist, and the working hardening rate is positive. The second segment starts from $\sigma_{c}$ and ends with $\sigma_{p}$, during which dynamic recrystallization exists and the working hardening rate is also positive. The third segment starts from $\sigma_{p}$ and ends with $\sigma_{ss}$, during which the working hardening rate is negative. Moreover, if we draw a tangent line at the critical point ($\sigma_{c}$, $\theta_{c}$), the intersection of the tangent line and σ axis is ($\sigma_{s}$, 0).

**Figure 8:**
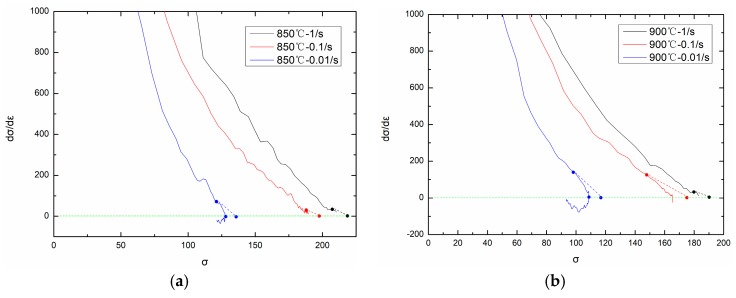

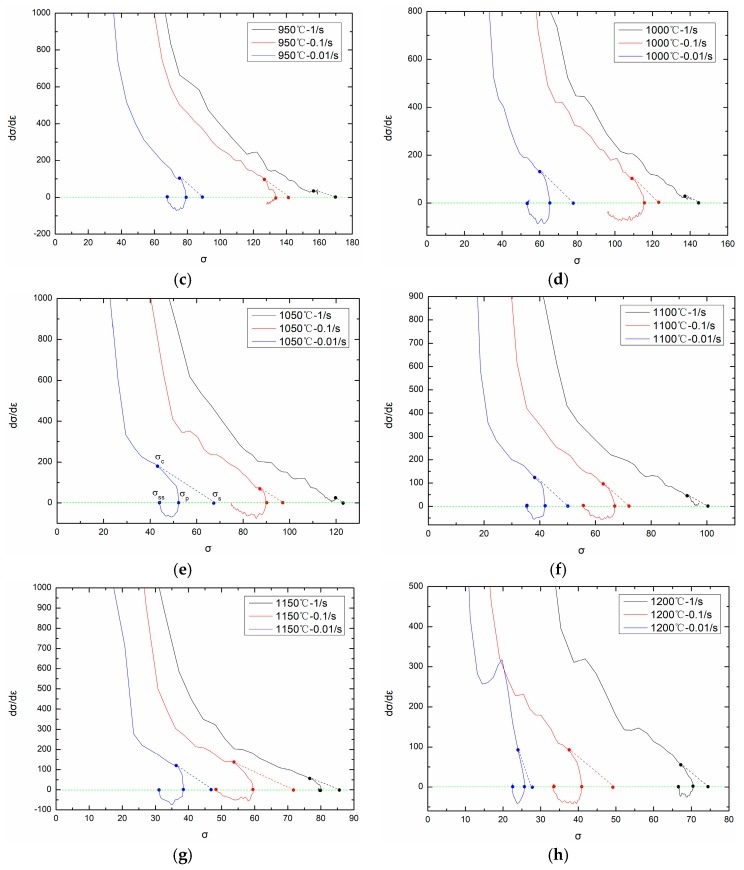
d*σ*/d*ε*-*σ* curves on different temperatures: (**a**) 850 °C; (**b**) 900 °C; (**c**) 950 °C; (**d**) 1000 °C; (**e**) 1050 °C; (**f**) 1100 °C; (**g**) 1150 °C; (**h**) 1200 °C.

Figure 8 shows the θ–σ curves under different temperatures and strain rates. From Figure 8, critical stress $\sigma_{c}$ on different temperatures and strain rates can be obtained (the second derivative, $d^{2}\theta /d\sigma^{2}=0$, when $\sigma =\sigma_{c}$). Based on experimental flow stress curves, the critical strain $\varepsilon_{c}$, corresponding to critical stress $\sigma_{c}$, can be easily obtained. By comparing critical strain $\varepsilon_{c}$ and peak strain $\varepsilon_{p}$ on each temperature and strain rate, the following relationship can be obtained:

(20) $$\varepsilon_{c}=0.83\varepsilon_{p}.$$

The saturation stress $\sigma_{s}$ can be obtained by the tangent line method mentioned above. The steady-state stress $\sigma_{ss}$ can be obtained from the experimental flow stress curves. The mathematical models of $\sigma_{s}$ and $\sigma_{ss}$ can be expressed as follows:

(21) $$\sinh(\alpha \sigma_{s})=kZ^{m},$$

(22) $$\sinh(\alpha \sigma_{ss})=kZ^{m},$$

**Figure 9:**
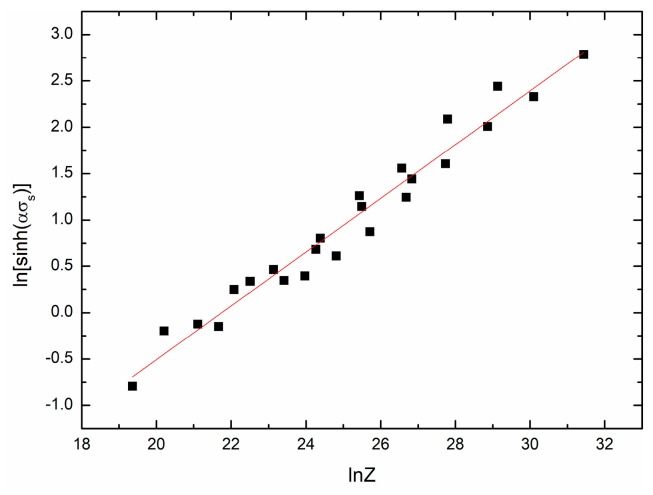
The relationship between $\ln[\sinh(\alpha \sigma_{s})]$ and lnZ.

**Figure 10:**
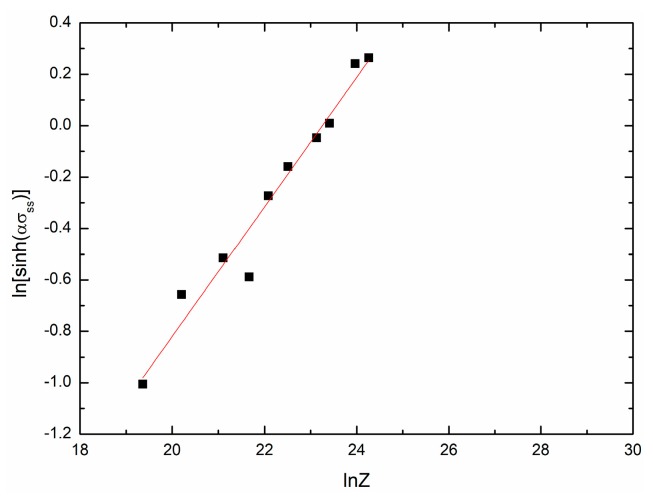
The relationship between $\ln[\sinh(\alpha \sigma_{ss})]$ and lnZ.

Figure 9 and Figure 10 show the fit results of $\ln[\sinh(\alpha \sigma_{s})]-\ln Z$ and $\ln[\sinh(\alpha \sigma_{ss})]-\ln Z$, respectively. The mathematical models can be given as:

(23) $$\sinh(0.01592\sigma_{s})=0.0018191Z^{0.29008},$$

(24) $$\sinh(0.01592\sigma_{ss})=0.0028439Z^{0.25212}.$$

**Figure 11:**
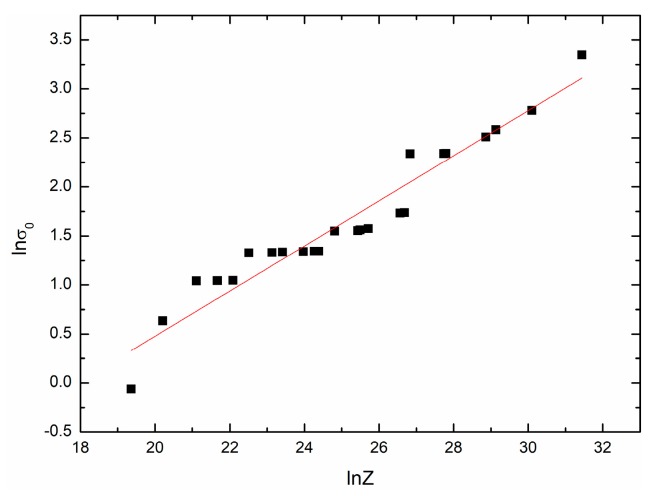
The relationship between $\ln\sigma_{0}$ and lnZ.

The mathematical model of initial stress $\sigma_{0}$ can also be obtained with the method mentioned above. Figure 11 shows the fit result. The following expression can be given:

(25) $$\sigma_{0}=0.016168Z^{0.23010}.$$

The constant $k_{2}$ on different temperatures and strain rates can be given by the following expression:

(26) $$k_{2}=\ln\frac{\sigma -\sigma_{s}}{\sigma_{0}-\sigma_{s}}(-2)/\varepsilon .$$

**Figure 12:**
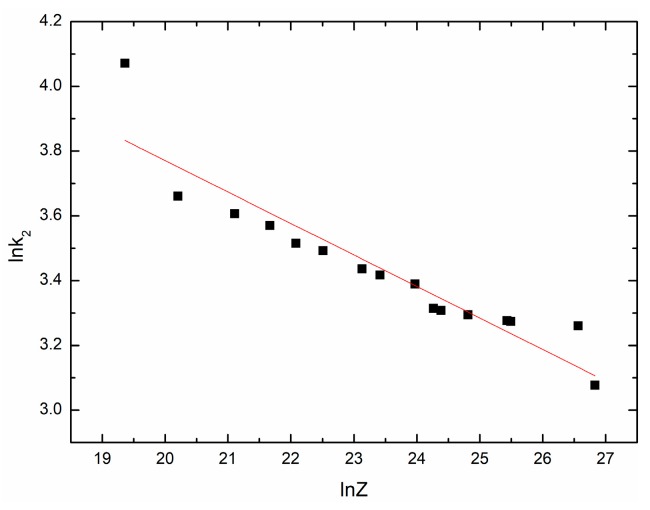
The relationship between $\ln k_{2}$ and lnZ.

Based on the stress-strain data ($\varepsilon \;＜\;\varepsilon_{c}$), many values of $k_{2}$ on each temperature and strain rate can be obtained, and the average value is taken. Figure 12 shows the fit result. The mathematical model is as follows:

(27) $$k_{2}=303.59103Z^{-0.097240}.$$

The volume fraction of DRX can be given as follows:

(28) $$X_{drx}=\frac{\sigma_{WH}-\sigma }{\sigma_{s}-\sigma_{ss}},\;\left(\varepsilon \geq \varepsilon_{c}\right).$$

**Figure 13:**
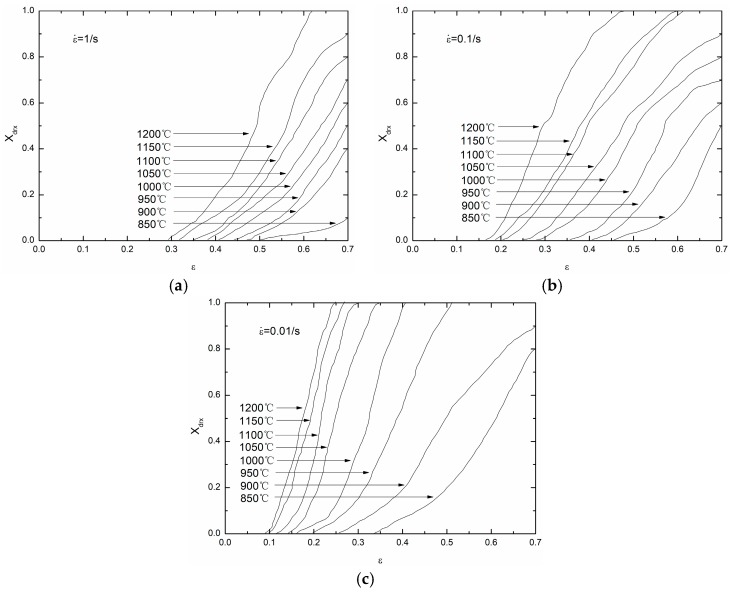
The $X_{drx}$–ε curves on different temperatures and strain rates: (**a**) 1 /s; (**b**) 0.1 /s; (**c**) 0.01 /s.

Based on Equation (28), different groups of ($X_{drx}$, ε) on each temperature and strain rate can be obtained. Figure 13 shows the $X_{drx}$–ε curves with different temperatures and strain rates.

Making some transformations and taking the natural logarithm on the kinetic model of DRX gives:

(29) $$\ln(-\ln(1-X_{drx}))=\ln k_{d}+n_{d}\ln(\frac{\varepsilon -\varepsilon_{c}}{\varepsilon_{p}}),\;\left(\varepsilon \geq \varepsilon_{c}\right).$$

**Figure 14:**
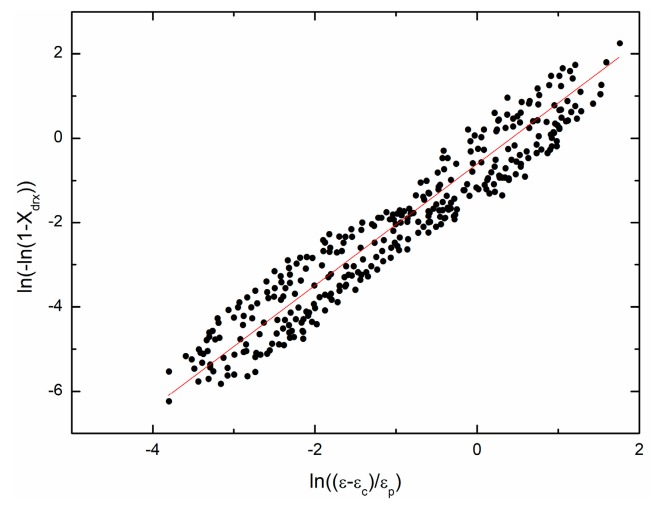
The relationship between $\ln(-\ln(1-X_{drx}))$ and $\ln(\frac{\varepsilon -\varepsilon_{c}}{\varepsilon_{p}})$.

Figure 14 shows the fit result; $n_{d}$ = 1.4414, $k_{d}$ = 0.5511 are obtained.

### 4.4. Verification of the Proposed Two-Stage Constitutive Model

**Figure 15:**
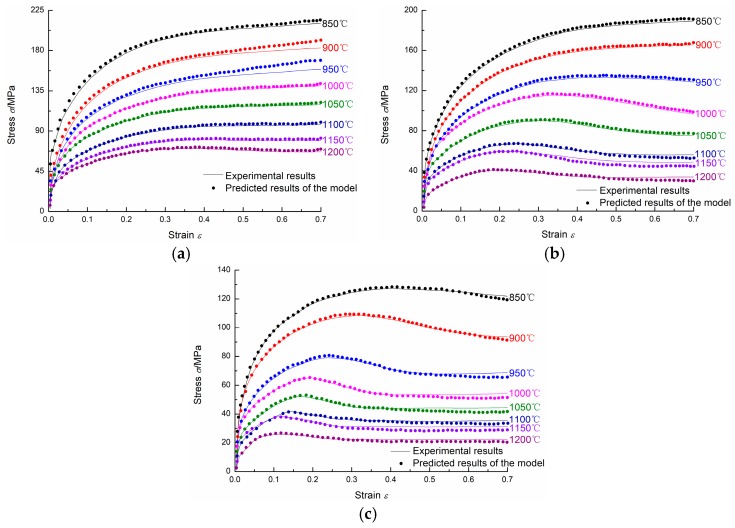
The comparison between experimental results and predicted results of the model: (**a**) 1/s; (**b**) 0.1/s; (**c**) 0.01/s.

Based on the activation energy Q and the material parameters ($\varepsilon_{p}$, $\varepsilon_{c}$, $\sigma_{s}$, $\sigma_{ss}$, $\sigma_{0}$, $k_{2}$, $n_{d}$, $k_{d}$) determined above, the flow stress under any temperature, strain rate, and strain can be calculated. Figure 15 shows the comparison between the experimental results and the predicted results of the model. The predicted flow stress values are in good agreement with the experimental flow stress values. Thus, the proposed two-stage constitutive model can give a reasonable estimate of the flow stress of 20Mn5 steel and can be used for numerical simulation of the hot forging of a 20Mn5 solid steel ingot.

In order to evaluate the accuracy of the proposed two-stage constitutive model, standard statistical parameters (correlation coefficient *R* and average absolute relative error AARE) are calculated. Correlation coefficient *R* can give us information on the strength of the linear relationship between the predicted flow stress and experimental flow stress. *R* and AARE are expressed as follows:

(30) $$R=\frac{\sum_{i=1}^{N}\left(\sigma_{E,i}-\bar{\sigma_{E}})(\sigma_{P,i}-\bar{\sigma_{P}}\right)}{\sqrt{\sum_{i=1}^{N}{\left(\sigma_{E,i}-\bar{\sigma_{E}}\right)}^{2}}\sqrt{\sum_{i=1}^{N}{\left(\sigma_{P,i}-\bar{\sigma_{P}}\right)}^{2}}},$$

(31) $$AARE=\frac{\sum_{i=1}^{N}\left|\frac{\sigma_{P,i}-\sigma_{E,i}}{\sigma_{E,i}}\right|}{N},$$

where $\sigma_{E,i}$ and $\sigma_{P,i}$ are the *i*-th experimental flow stress and predicted flow stress, respectively; $\bar{\sigma_{E}}$ and $\bar{\sigma_{P}}$ are the average value of the experimental flow stress and predicted flow stress, respectively; *N* is the number of the experimental flow stress.

The calculated *R* is 0.991, which shows that the strength of the linear relationship between the experimental flow stress and predicted flow stress is very strong. The calculated AARE is 3.01%, which indicates that the prediction accuracy of the proposed two-stage constitutive model is very high and the proposed two-stage constitutive model can be used for numerical simulation of hot forging of 20Mn5 solid steel ingot.

## 5. Conclusions

(1) During hot compression of 20Mn5 steel, firstly, the flow stress increases to a peak stress, and then the flow stress decreases gradually to a steady-state stress with the increase of strain. Peak stress does not occur at 850–1100 °C and 1/s, or at 850–900 °C and 0.1/s. At a higher temperature and lower strain rate, the peak strain, steady-state strain, peak stress, and steady-state stress are smaller.
(2) The material constants (α, n, Q, A) are calculated based on the peak stress.
(3) A two-stage constitutive model of 20Mn5 steel is established. The related material parameters ($\varepsilon_{p}$, $\varepsilon_{c}$, $\sigma_{s}$, $\sigma_{ss}$, $\sigma_{0}$, $k_{2}$, $n_{d}$, $k_{d}$) are determined.
(4) The proposed constitutive model can give a good prediction of the flow stress on different temperatures, strain rates, and strains, and can be used for numerical simulation of the hot forging of a 20Mn5 solid steel ingot.

## Figures and Tables

**Table 1:** Chemical composition of 20Mn5 steel (wt %).

C	Si	Mn	P	S	Cr	Ni	Mo	Al
0.24	0.26	1.47	0.0084	0.0020	0.15	0.076	0.020	0.015
